# To the Profession

**Published:** 1852-05

**Authors:** 


### EDITORIAL.

*To the Profession.*

THIS being the first number of a new series of the *North Western Medical and Surgical Journal*, it seems the proper time for its present editors to make a brief exposition of their plan for its future publication, in order that its readers may know what to anticipate hereafter from a periodical which, under the superintendence of its recent able editor has already acquired character, the prospect of permanence, and a large number of patrons.

The change from a bi-monthly to a monthly journal, has been made in compliance with the expressed wishes of a large number of its patrons, and in order that its readers may receive oftener and more promptly notices of new discoveries and other interesting and important medical intelligence.

Each number will contain hereafter communications, selections, and editorials in the order as above named.

The part devoted to ORIGINAL COMMUNICATIONS will be made up of reports of interesting cases, important facts observed, and other matters of interest to the practitioner, such as will be furnished by the numerous contributors to this journal in various parts of the great North-West; who will doubtless continue their favors without further solicitation, knowing, as they must, that it is upon them that we rely principally for the means of rendering this department useful and interesting to physicians.

In making SELECTIONS, especial regard will be had to the wants of practitioners, and to the importance of well established facts as compared with mere hypotheses and theoretical opinions. In addition to extracts from American and English medical journals, the eclectic department will contain also, from time to time, translations from French and German periodicals.

Under the head of EDITORIALS, will be included all reviews, notices, and abstracts of recently published books, papers, and periodicals, in which it will be the object of the editors to give, frequently, in addition to a brief synopsis of their contents, facts and information derived from other sources, which may have a bearing upon the subject under consideration. This department will contain, also, short accounts of the proceedings of medical associations and societies, and all other interesting medical intelligence of whatever nature; and, finally, the results of the investigations and observations made by the editors themselves, for which the two hospitals now open in Chicago, furnish unusual facilities.

Believing as we do, that this, the oldest medical periodical in the North-West, will, if conducted upon the above plan, continue to receive the patronage and support of north-western physicians, we renew our editorial labors in confidence and with bright anticipations for the future. H.

